# Time in mind: a multidisciplinary review on temporal perception, cognition, and memory

**DOI:** 10.3389/fcogn.2025.1688754

**Published:** 2026-01-21

**Authors:** Jeffery R. L. Pendleton, Nicola S. Clayton

**Affiliations:** Department of Psychology, University of Cambridge, Cambridge, United Kingdom

**Keywords:** comparative cognition, episodic memory, episodic-future thinking, episodic-like memory, linguistic relativity, mental time travel, mind time, temporal cognition

## Abstract

This review examines temporal cognition through the lens of Mental Time Travel (MTT): the subjective experience of recalling past events and using them to construct future scenarios. The analysis specifically addresses how language and cultural context affect these abilities, integrating psychology, linguistics, cognitive neuroscience, and anthropology. Findings from comparative cognition challenge whether they are uniquely human. Although such an approach was traditionally taken in non-human primates, the field of comparative cognition has become much more diverse. Comparative insights derived from studies of corvid and cephalopod cognition are particularly pertinent, as they suggest these abilities have evolved more widely within the animal kingdom, especially in groups with very different neural architectures, raising questions about whether these abilities have evolved convergently in species undergoing similar selection pressures or independently in those subject to different selection pressures, as opposed to homologous evolution widespread amongst these animal taxa. These evolutionary perspectives inform theories of human temporal cognition and Mental Time Travel, influencing memory encoding and retrieval processes, false memory production, as well as the mechanisms underlying temporal cognition, such as episodic memory formation, interval timing, and circadian modulation of memory consolidation. Additionally, the review evaluates evidence on the cognitive impact of technological tools (calendars, clocks, and other technologies) used to externalize and standardize temporal frameworks, including implications for subjective perception and memory accuracy, and identifies directions for future interdisciplinary research. Building on this synthesis, we advance five core claims: that elements of temporal cognition likely arise under convergent evolutionary pressures; that language, culture, and social organization tune how people represent and use time; that technologies which externalize time can reshape behavior by aligning with or pulling against internally constructed event time; that memory is adaptively biased toward flexible, future-oriented construction rather than veridical record; and that these processes are structured by “mind time” and extended via transpersonal extended mental time travel, whereby shared representations support the projection and coordination of futures across individuals and generations.

## 1 Introduction

The human experience of time is a paradox. Physical time is a fundamental dimension of the universe that is linear and measured objectively by atomic clocks and astronomical cycles. Psychological time, however, is a fluid and subjective construction; it can stretch during moments of fear or boredom and compress during periods of engagement and joy (Droit-Volet and Meck, 2007). A combination of neural processes, experience, linguistics, cultural systems, and our shared evolutionary history allows for this “mind time”, a term we introduce to represent the subjective-dynamic temporal experience. In contrast to unidirectional physical time, it allows for bi-directional Mental Time Travel (MTT), the ability to mentally project oneself backwards in time to re-experience personal past events and forwards to pre-experience possible future events (Suddendorf and Corballis, 1997). Mental time travel is bidirectional but asymmetric in phenomenology and control demands, which matters for how linguistic, cultural, and technological contexts interact with mind time. The bidirectionality differs however: with retrospective MTT we jump back to specific moments in time then play those memories forward whereas prospective MTT is a gradual unfolding of the future (Clayton and Wilkins, 2018). This review uses the prospective brain hypothesis (Schacter et al., 2007, 2008) as a unifying framework to argue that the primary adaptive function of memory is providing the raw material for future simulations, a thesis that explains phenomena across neuroscience, linguistics, and comparative cognition.

MTT consists of two fundamental components: a retrospective capacity that relies on episodic memory, and a prospective capacity that supports foresight and planning for the future (Suddendorf and Corballis, 1997). The retrospective component, episodic memory, enables the conscious recollection of specific personal experiences, enriched with contextual details and accompanied by the sense of re-experiencing the original event (Tulving, 1985; Clayton et al., 2007). This phenomenology sets it apart from semantic memory, which stores decontextualised world knowledge, explained by Tulving's Remember-Know distinction (Tulving, 1985). For instance, one might know the fact that Paris is the capital of France but remembering when one learnt this fact involves mentally re-experiencing the event. Recalling the context of a memory's acquisition (the sights, sounds, and feelings of that moment in the classroom) is known as source memory, a component of episodic memory (Johnson et al., 1993).

This self-aware navigation (Clayton and Wilkins, 2017) through subjective time depends on a form of consciousness that Tulving (1985) termed *autonoetic* (“self-knowing”), an awareness of oneself as a continuous entity through time, recognizing that the self who experienced a past event is the same self who exists in the present and will exist in the future. This episodic remembering also entails *authorship* and *ownership*: a remembered episode is indexed to one's *own* past and experienced as “mine” and is essential to genuine memory, which must be “dated” in one's personal past (James, 1950). This self-knowing, self-owned awareness enables an individual to judge that a past event occurred and to mentally relive it from a first-person perspective.

The metaphor of “travel” is appropriate: it implies a traveler (the self), a landscape (a mental timeline), and a capacity for voluntary movement (projection) (Wilkins and Clayton, 2017). Tulving later introduced *chronesthesia* to denote the subjective awareness of temporal relations, the sense of being “in a sea of time” and of locating events as before, after, or now (Tulving, 2002a,b). Building on this, “mind time” is used here to emphasize the agentic, self-modulated navigation of that temporal landscape: as the self changes, so too does the felt structure of subjective time, echoing Lewis Carroll's observation, “I can't go back to yesterday—because I was a different person then” (Carroll, 1865, 2000, p. 155). Framed in this way, MTT is elevated from a mere memory function to a higher-order cognitive faculty in which autonoetic consciousness, chronesthesia, and imagination jointly support self-aware projection across past, present, and future, guiding the traveler through mind time.

From an evolutionary standpoint, the value of any cognitive faculty is measured by its contribution to future survival and reproduction. Natural selection acts on traits that enhance future fitness, not on the fidelity of a historical record *per se* (Suddendorf and Corballis, 2007). An organism's survival depends on its ability to anticipate future needs and threats by dissociating the present in order to act accordingly. Humans, however, show a “presentism” in affective forecasting; they project current preferences, emotions, and contexts into imagined futures, so we expect tomorrow to resemble today more than it ever will (Gilbert and Wilson, 2007; Gilbert, 2006). From this perspective, the ability to flexibly recombine elements of past experiences to construct novel simulations of what might happen in the future confers a significant selective advantage (Klein et al., 2010). Memory's apparent imperfections, such as its proneness to error and distortion, are better understood as adaptive trade-offs. This view reframes the “seven sins of memory”, further explained in a later section, (Schacter, 2001) by treating them as the consequences of a system that values cognitive flexibility over rote recall (Schacter, 2001; Schacter et al., 2008). It aligns with the ‘Janus hypothesis,' which posits that remembering the past and imagining the future are two sides of the same cognitive coin, reliant on shared neural machinery (Suddendorf and Corballis, 2007). This evolutionary rationale also explains the ‘seven myths of memory,' which are deconstructed as misconceptions that arise precisely because our minds evolved to anticipate the future (Clayton and Wilkins, 2018).

A comprehensive understanding of “mind time” requires drawing on multi-disciplinary perspectives. Cognitive neuroscience helps to identify the shared neural substrates for remembering the past and imagining the future. Linguistics and anthropology explain how cultural models and linguistic conventions inform temporal reasoning. Comparative cognition offers an evolutionary context by examining the origins of these capacities, while experimental psychology characterizes memory's core mechanisms, including its malleability and modulation.

We advance five claims and organize the paper around them. First, elements of temporal cognition likely arise under convergent pressures across species, although the precise evolutionary pathways remain open. Second, language, culture, and social organization tune how people represent, talk about, and use time in everyday reasoning. Third, technologies that externalize time, including clocks, calendars, and digital platforms, can reshape behavior by aligning with or pulling against internally constructed event time. Fourth, the primary adaptive function of memory is to supply flexible material for future simulation, so some error patterns reflect trade-offs of a system optimized for constructive use rather than verbatim record (Schacter, 2001, 2012). Fifth, we introduce mind time to refer to the internally constructed, event-relative temporal structure used to recall and introduce transpersonal extended mental time travel (teMTT) to describe how communication and shared representations allow people to project and coordinate beyond the individual, across people and across generations.

In Section 2 we outline the neural foundations of constructive remembering and simulation. In Section 3 we examine how linguistic practices and cultural conventions bias temporal orientation and judgment. In Section 4 we consider evolutionary constraints and opportunities that can yield similar temporal solutions under different architectures. In Section 5 we bring together key modulators of temporal accuracy that matter for real-world use. In Section 6 we contrast mind time with externalized clock time, develop the idea of teMTT, and draw practical implications.

**Figure 1:**
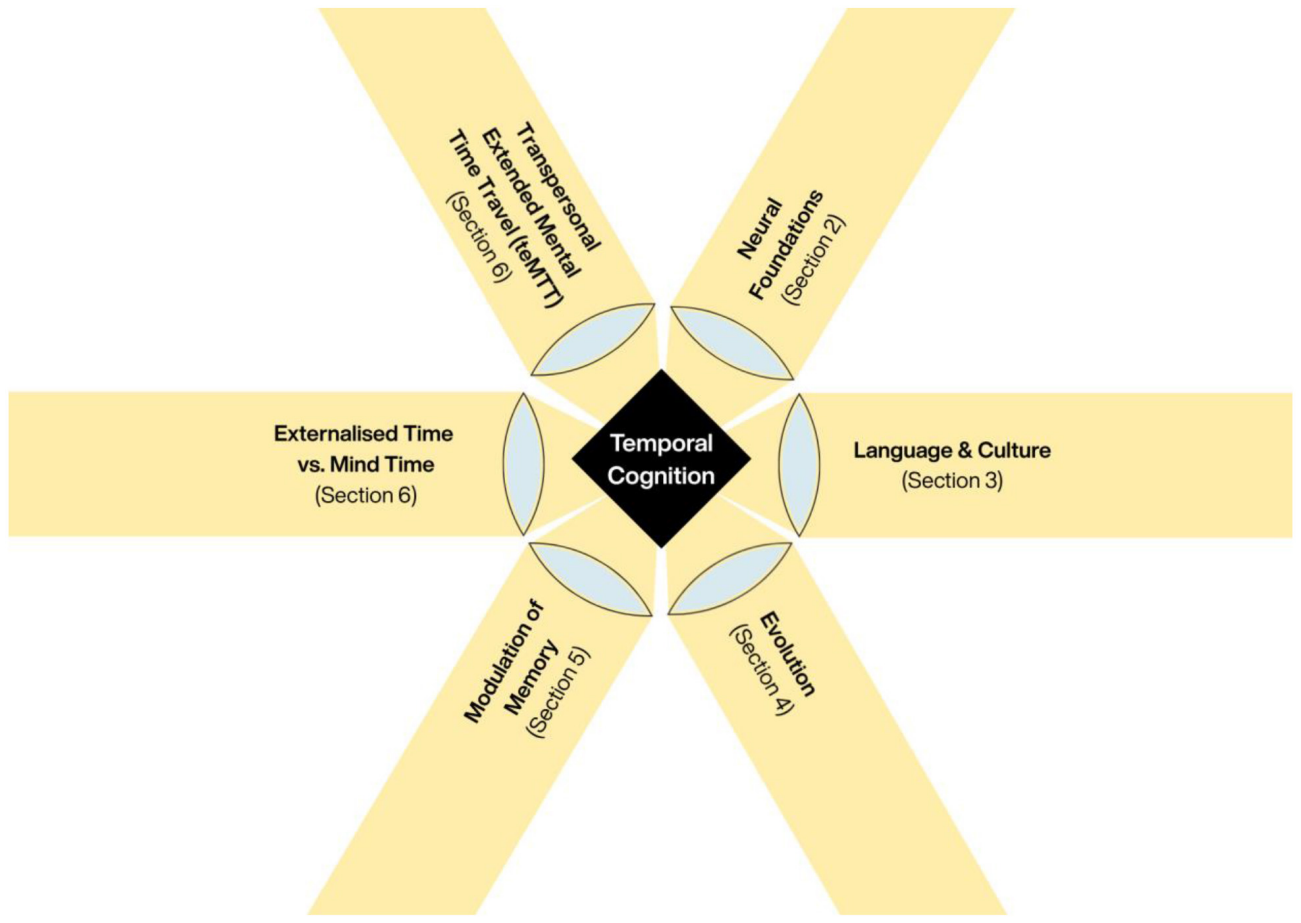
Conceptual overview of the review's organizing framework. Temporal cognition is positioned as the central construct, with six contributing and interacting components arranged around it: neural foundations, language and culture, evolutionary accounts, modulatory influences on memory, externalized time vs. “mind time,” and transpersonal extended mental time travel (teMTT). The figure is introduced and described at the end of Section 1. For detailed explanation and interpretation, please see the main text.

A cross-disciplinary overview that places neural foundations, language and culture, evolution, modulation of memory, externalized time vs. mind time, and transpersonal extended mental time travel (teMTT) around temporal cognition appears in Figure 1.

We treat retrospective remembering and prospective simulation as expressions of a largely shared constructive system with content-dependent specializations, consistent with classic accounts that emphasize common operations for recollection and prospection in hippocampal–cortical networks (Tulving, 1985; Buckner and Carroll, 2007; Schacter and Addis, 2007). At the same time, we acknowledge selective dissociations tied to control, monitoring, and task demands that can differentially weight component processes without implying distinct dedicated systems (Dudai, 2002; Schacter et al., 2012). In Section 2 we return to these overlap-vs. -dissociation proposals attributed to Tulving, Schacter, and Dudai, and we relate them to contemporary predictive-processing views in which generative models support both memory reconstruction and future simulation through similar inferential machinery (Friston, 2010; Clark, 2013).

## 2 Neural foundations of mental time travel

The idea that remembering the past and imagining the future are closely related is encapsulated in the Constructive Episodic Simulation Hypothesis (Schacter et al., 2008; Schacter and Addis, 2007). This hypothesis posits that the primary function of the episodic memory system is to provide a repository of details that can be flexibly extracted and recombined to simulate novel future events, rather than to create a veridical, high-fidelity record of the past (Schacter and Addis, 2007; Addis et al., 2009; Schacter et al., 2008). This constructive capacity is highly adaptive, as it allows individuals to anticipate and plan for future scenarios (Szpunar, 2010). It also provides a principled explanation for why memory is so often prone to error and distortion; a system optimized for flexible recombination is inherently less suited for rote reproduction (Schacter, 2001).

**Figure 2:**
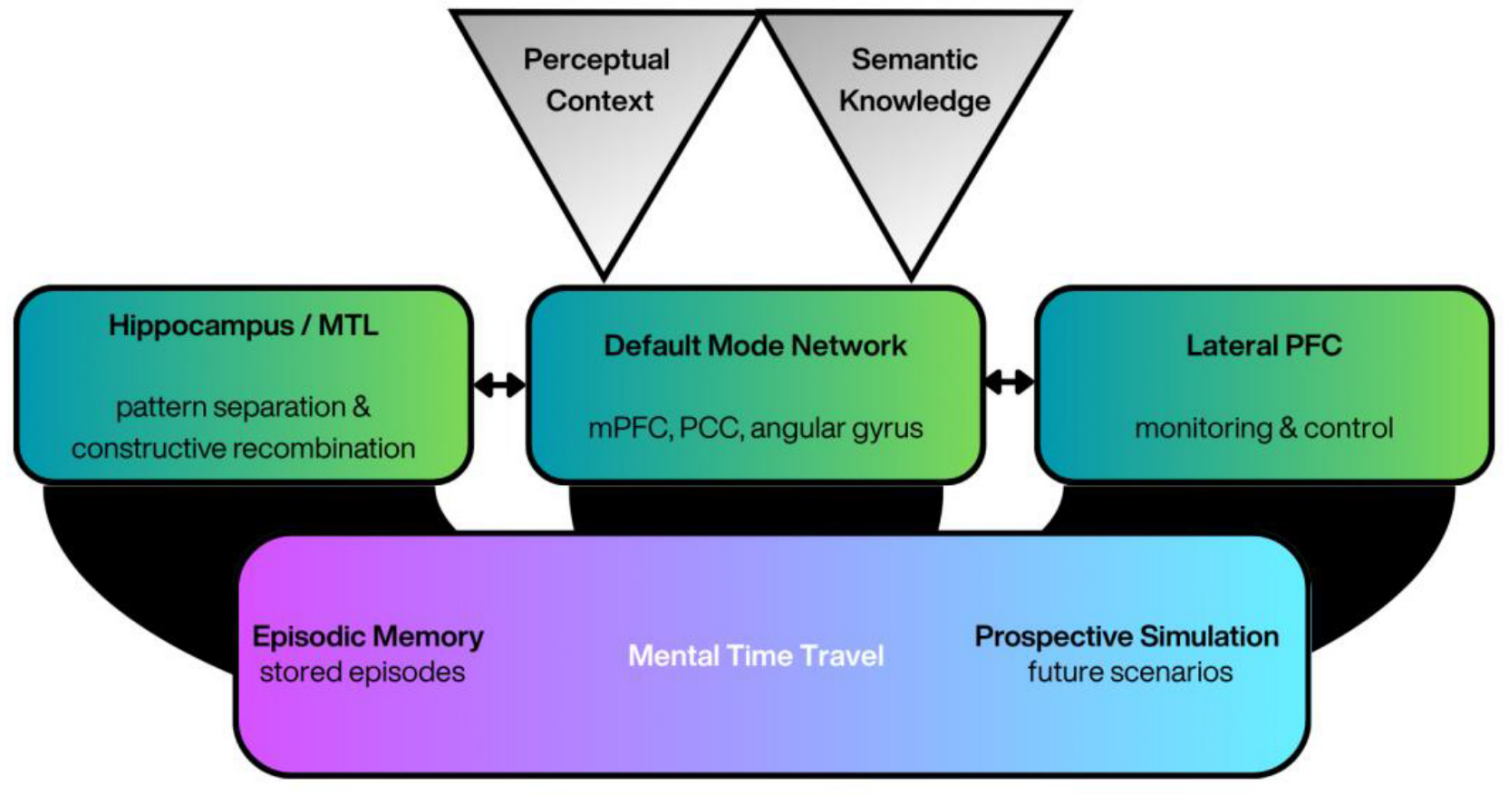
Neural systems supporting constructive remembering and prospective simulation. Schematic summary of how hippocampus/medial temporal lobe (MTL) mechanisms (pattern separation and constructive recombination) interact with the default mode network (including mPFC, PCC, and angular gyrus) and lateral prefrontal cortex (monitoring and control), drawing on perceptual context and semantic knowledge to support episodic memory retrieval and prospective simulation of future scenarios. This figure is introduced in Section 2. For detailed explanation and interpretation, please see the main text.

This review pairs systems-level human evidence for this constructive system with rodent circuit-level mechanisms that specify candidate codes for simulation. The core network spans hippocampal and medial temporal structures, default mode cortical hubs, and lateral prefrontal monitoring. As we will see, it supports both retrospective and prospective use with content-dependent specializations rather than strict future specificity (Addis et al., 2007; Buckner and Carroll, 2007; Buckner et al., 2008). Figure 2 provides a schematic overview of this core network, showing how hippocampal and medial temporal structures, default mode cortical hubs, and lateral prefrontal regions interact with perceptual context and semantic knowledge to support mental time travel between stored episodes and prospective simulations.

Both rodent and human work identifies prospective codes in hippocampal–entorhinal circuits that support constructive mental time travel. During pauses in movement and during sleep, hippocampal ensembles compress sequences as replay and preplay, including forward progressions that recapitulate recent trajectories and reverse sequences that trace back from outcomes to earlier states (Diba and Buzsáki, 2007; Dragoi and Tonegawa, 2011). Immediately before goal-directed runs, place-cell sequences can depict candidate future paths from the current location toward a remembered goal, providing a circuit mechanism for evaluating alternatives (Pfeiffer and Foster, 2013). In humans, intracranial and fMRI studies indicate grid-like coding and replay-like dynamics that generalize to imagined navigation and planning, and even to abstract, non-spatial knowledge structures (Doeller et al., 2010; Horner et al., 2016; Constantinescu et al., 2016; Liu et al., 2019). Across species, hippocampal and entorhinal populations also carry goal-related information, including vector-like organization that points toward current targets in rodents and single-neuron responses to remote spatial goals in humans (Hok et al., 2007; Ormond and O'Keefe, 2022; Tsitsiklis et al., 2020). These codes provide mechanisms for compressing, reordering, and comparing candidate trajectories that underwrite constructive simulation.

Neuroimaging studies using functional magnetic resonance imaging (fMRI) have been instrumental in identifying the neural substrates of this “prospective brain” (Schacter et al., 2007). These studies consistently reveal a significant overlap in brain activation when individuals remember past autobiographical events and when they imagine future ones (Schacter et al., 2007, 2008). This “core network” includes the hippocampus and adjacent medial temporal lobe structures, the medial prefrontal cortex (mPFC), lateral temporal cortex, and posterior regions such as the praecuneus and retrosplenial cortex (Schacter et al., 2007; Addis et al., 2007; Buckner and Carroll, 2007). The engagement of this network across both tasks supports the hypothesis that a common set of processes is involved in both retrospective and prospective thought (Maguire, 2001). This network shows a significant overlap with the brain's default mode network (DMN), a set of regions that are most active during periods of rest and internally directed thought, such as mind-wandering, and show decreased activity during externally focused tasks (Buckner et al., 2008). The correspondence between the MTT network and the DMN suggests that our capacity to mentally travel in time may have emerged from a more general system dedicated to creating and manipulating internal mental models of the world, detached from immediate sensory input.

Further research has revealed important distinctions within the shared activity of the core network, particularly by separating the cognitive processes of event construction from event elaboration. In a study by Addis et al. (2007), participants were asked to construct a past or future event in response to a cue and then, after indicating they had the event in mind, to elaborate on its details. While conjunction analyses confirmed that the left hippocampus was commonly engaged during the construction of both past and future events, this initial phase also showed considerable neural differentiation. Imagining future events uniquely recruited the right frontopolar cortex, a region associated with prospective thinking, and the right hippocampus, possibly in response to the novelty of constructing a new event representation (Addis et al., 2007). In contrast, the subsequent elaboration phase was characterized by a remarkable degree of neural overlap across past and future conditions, particularly in regions associated with self-referential processing and contextual imagery. This suggests that once an event scaffold is constructed, the addition of sensory and emotional detail engages the same mental simulation machinery, regardless of the event's temporal location (Addis et al., 2007).

Complementing the constructive simulation hypothesis, the Self-Projection Hypothesis proposes that the core network's most fundamental function is to enable a shift in perspective away from the immediate present (Buckner and Carroll, 2007). This capacity for “self-projection” allows an individual to mentally place themselves in an alternative context; a different time (past or future), a different place (spatial navigation), or inside another person's mind (theory of mind). According to this view, MTT is one specific manifestation of a more general-purpose simulation ability supported by the core network (Buckner and Carroll, 2007). This framework helps to explain the consistent co-activation of this network across tasks that appear quite different but share the common requirement of constructing a scene or perspective separate from immediate sensory reality.

An open issue is whether the increased activity observed during future thinking is specific to prospection or reflects a more general demand of imagining any novel episodic event. To address this, Addis et al. (2009) designed an experiment in which participants were required to imagine both future and past events, by recombining details from their actual experiences. This design prevented participants from simply recasting a whole past memory as a future one. Using spatiotemporal partial-least-squares, the researchers identified two distinct subsystems within the core network. One subsystem, which included the posterior hippocampus, parahippocampal gyrus, and visual cortex, was preferentially engaged when remembering actual past events, consistent with its role in retrieving rich visuospatial and contextual detail. A second, more extensive subsystem, including the anterior hippocampus and mPFC, was preferentially engaged when imagining events, regardless of whether they were placed in the past or the future (Addis et al., 2009). This finding suggests that some regions previously thought to be future-specific are part of a general-purpose “imagination engine” responsible for the flexible recombination of memory elements into a coherent, novel scenario (Addis et al., 2009).

This model is expanded upon by work on the cognitive control of memory by Simons and colleagues. Their research demonstrates that the successful construction of a novel mental event requires crucial post-retrieval mechanisms for monitoring and evaluating the recombined information (Simons and Spiers, 2003). A key brain region for this function is the rostrolateral prefrontal cortex (RLPFC), which plays a vital role in reality monitoring, the ability to distinguish between memories of real experiences and those of imagined ones (Simons et al., 2017). This evaluative capacity is supported by a wider network including the dorsolateral PFC and the angular gyrus, with the latter being particularly important for the rich, first-person subjective experience of remembering (Rissman et al., 2010; Simons et al., 2022). The hippocampus, in particular, is thought to be central to this recombination process due to its well-established part in relational memory, the binding of disparate elements of an experience into a coherent whole (Eichenbaum and Cohen, 2001). The anterior hippocampus seems specialized for this flexible, constructive binding, whereas the posterior hippocampus is more involved in the high-fidelity retrieval of established spatial and contextual frameworks.

The construction of these novel scenarios draws on more than episodic details. Semantic memory, our general knowledge about the world, its scripts, and schemas, organizes and contextualizes specific episodic details (Irish and Piguet, 2013). This idea is formalized in the semantic scaffolding hypothesis, which posits that semantic memory provides the conceptual structure or “scaffold” necessary to build a coherent simulation of a future event. For example, to imagine a future birthday party, one must first access the semantic schema for “birthday party” (e.g., it involves cake, guests, presents). This schema then guides the retrieval and integration of specific episodic details (e.g., the taste of a specific cake from a past party, the face of a friend who will be invited). Patients with semantic dementia, who have degraded conceptual knowledge but relatively preserved recent episodic memory, show marked impairments in their ability to imagine the future, describing events that are fragmented and implausible (Irish and Piguet, 2013). This illustrates that both memory systems are necessary: semantic memory provides the generic structure, and episodic memory supplies the specific, self-relevant details that give simulations their richness and personal significance. The medial prefrontal cortex (mPFC) is thought to be involved in integrating these two streams of information, linking the simulation to one's personal goals and self-concept (D'Argembeau et al., 2008). Evidence from developmental and comparative studies also supports the interdependence of these systems in episodic foresight. For instance, Tulving's “Spoon Test” (Wilkins and Clayton, 2019) captures the essence of this integration by requiring individuals to recall a specific past episode, imagine a possible future scenario, and take appropriate action in the present to prepare for that future. Tulving emphasized that this preparation must be for a need arising in a different place or context, demonstrating transfer of past knowledge to a novel setting (Tulving, 2005). Performance on such tasks accentuates how semantic frameworks guide retrieval while episodic memory provides the subjective, autonoetic detail necessary for projecting oneself forward in time. This neural data support the view that construction and monitoring are shared operations that are repurposed for prospection, and they motivate the analysis in Section 3 of how language and culture steer temporal attention and interpretation.

## 3 The lens of language and culture

We use language and culture as a lens on how communities set default temporal orientation, disambiguate reference, and bias interpretation in ways that are modest in size yet reproducible across tasks. While the neural basis for MTT may be a universal feature of human cognition, the way this capacity is organized, expressed, and habitually deployed can be influenced by the cognitive tools provided by language and culture. The linguistic relativity hypothesis, most famously associated with Whorf (1956), proposes that the language one speaks can influence patterns of thought. The strong, deterministic formulation of this idea, that language creates inescapable cognitive “prisons”, has been largely rejected. However, a substantial body of contemporary research supports a more moderate view: language influences thought by providing recurrent patterns of expression which, through repeated use, become habitual patterns of attention and conceptualization (Gentner and Goldin-Meadow, 2003). The domain of time, being abstract and imperceptible, is particularly susceptible to such influences. Because time cannot be directly observed, it is often structured through metaphor and linguistic convention, making it an ideal case study for exploring how language shapes cognition (Boroditsky, 2001).

One influential framework for understanding this relationship is Slobin's (1996) “thinking for speaking” hypothesis. This account suggests that in preparing to speak, individuals must attend to the specific distinctions and categories that their language encodes. For example, English grammar obliges speakers to choose whether to frame an action as ongoing (“he is running”) or as a general habit (“he runs”) through the use of progressive aspect, whereas German does not grammatically require such a distinction; *er läuft* can refer to both situations, with context and optional adverbs supplying the temporal detail. Over time, such obligatory grammatical choices in English may lead speakers to habitually track the temporal unfolding of events, while German speakers may focus less on boundedness or ongoingness unless it is communicatively relevant.

In this view, language does not rebuild cognition from the ground up, it systematically tunes attention to certain temporal features, subtly guiding the mental habits we carry into non-linguistic thought.

### 3.1 Spatial metaphors and mental timelines

Across the world's languages, time is conceptualized through spatial metaphors (Lakoff and Johnson, 1980). We speak of a long time, of moving deadlines forward, and of putting the past behind us. However, the specific spatial axes used are not universal, and these linguistic differences correlate with differences in non-linguistic temporal reasoning. A more formal way to analyse these spatial conceptions is through the lens of Frames of Reference (FoRs), a system originally developed for space (Levinson, 1996) and later applied to time (Bender et al., 2010). A FoR specifies how the location of a figure (an event) is determined relative to a ground (a reference event). An intrinsic FoR uses the inherent facets of the ground object (e.g., “the meeting is at the back of the week”). An absolute FoR uses fixed external bearings, like cardinal directions (e.g., “time flows eastward”). A relative FoR depends on the viewpoint of an observer (e.g., “the past is in front of me”). Linking back to “mind time”, FoRs set the default orientation of the internal timeline, whether the self is treated as moving through time or time as moving past the self. This orientation biases chronesthetic judgements (e.g., which direction is taken as “future”) and performance on tasks that require projecting events forward or backward.

The most well-documented example of linguistic influence is the contrast between horizontal and vertical representations of time. In English, time is predominantly discussed using horizontal terms, reflecting a relative FoR where the observer's front/back axis is mapped onto the future/past. This linguistic pattern has cognitive consequences. In a series of seminal experiments, Boroditsky (2001) used a spatial priming paradigm to show that English speakers were faster to verify temporal propositions (e.g., “March comes before April”) after being primed with a horizontal spatial stimulus than a vertical one. In contrast, Mandarin Chinese speakers, whose language uses both horizontal and vertical spatial metaphors for time [e.g., *shàng ge yuè* (上个月), “up month,” for last month; *xià ge yuè* (下个月), “down month,” for next month], showed the opposite pattern. They were faster to make temporal judgments after a vertical prime (Boroditsky, 2001). This effect persisted even when Mandarin-English bilinguals were tested in English, and the strength of the vertical bias was correlated with the age at which they had learned English, suggesting that the patterns of one's native language can establish enduring cognitive habits (Boroditsky, 2001).

Another organizing principle is writing direction. In a non-linguistic task, Fuhrman and Boroditsky (2010) asked participants to arrange a series of pictures depicting a temporal progression (e.g., a banana being eaten). English speakers, who read and write from left to right, consistently arranged the cards from left to right. Hebrew speakers, whose script runs from right to left, arranged the cards from right to left (Fuhrman and Boroditsky, 2010). This spatial-temporal mapping appears to be automatic. In a reaction-time task, English speakers were faster to judge that an event was earlier when the response bank was on their left, whereas Hebrew speakers were faster when the earlier bank was on their right. This interference supports that people automatically and implicitly access these culturally specific spatial representations when thinking about time, even in the absence of language (Fuhrman and Boroditsky, 2010).

The conventional metaphor in many cultures places the future in front of the body and the past behind, often mirroring the experience of forward locomotion. However, this mapping is not universal. Speakers of Aymara, an indigenous language of the Andes, conceptualize the future as behind them (*qhipa pacha*, “back time”) and the past as in front (*nayra pacha*, “front/eye time”) (Núñez and Sweetser, 2006). This orientation is grounded in an epistemic metaphor: the past is known and has been seen, so it lies in the visual field ahead; the future is unknown and unseen, so it lies behind one's back. These temporal mappings appear in gesture, Aymara speakers point forward when speaking of the past and backward when speaking of the future (Núñez and Sweetser, 2006). A similar “past-in-front” conception has been described for Māori speakers in New Zealand, who describe themselves as walking backwards into the future, guided by the visible lessons of the past (Barlow, 1994). Such cases show that the directionality of mental timelines can be culturally contingent, shaped by whether the dominant metaphor for time is based on motion through space or on the limits of visual perception (Bender et al., 2010).

### 3.2 Asymmetry in forward and backward projection

In the physical world, time's arrow is unidirectional, driven by the increase of entropy. MTT, however, is bidirectional: we can project backwards to re-experience the past and forwards to pre-experience possible futures (Tulving, 2002a,b; Schacter et al., 2007; Corballis, 2013; Clayton and Wilkins, 2018). These two operations are not cognitively symmetrical. Episodic remembering typically begins by “jumping” to a specific, known moment, rich in spatiotemporal and sensory detail, and then replaying events in their original forward order (Schacter and Addis, 2007). By contrast, episodic simulation of the future tends to unfold gradually from the present into an indeterminate domain, where multiple possible trajectories compete and outcomes remain uncertain (Schacter et al., 2012).

This asymmetry has at least two implications. First, past events, even if partial or distorted (Bartlett, 1932; Loftus, 2005), often feel more certain than the future because they are anchored in autonoetic consciousness, a subjective sense of “having been there” (Tulving, 2005). Second, the cognitive operations differ: re-experiencing the past draws on stored event sequences optimized for forward replay, whereas reversing well-learned verbal or motor sequences (e.g., reciting the alphabet backwards or performing a dance in reverse) is markedly more error-prone (Zacks and Tversky, 2001; Rosenbaum et al., 2007). This forward bias likely reflects the way episodic memory encodes temporal information for action planning and prediction (Kurby and Zacks, 2008).

From a comparative cognition perspective, this forward asymmetry is not uniquely human. In scrub-jays, integrated what–where–when (WWW) representations support both retrospective memory-based recovery of past caches and prospective adjustment of future caching decisions, indicating that the same declarative content can be redeployed for forward planning without ruling out reconstructive use of the past (Clayton and Dickinson, 1998; Clayton et al., 2001a; de Kort et al., 2005; Raby et al., 2007). In humans, the same forward-oriented sequencing underpins both retrospective recall and prospective simulation, but the latter must contend with the “fuzziness” of an open-ended future. Although both forms of MTT recruit overlapping neural substrates (Addis et al., 2007; Schacter et al., 2012), their differing demands (certainty vs. uncertainty, replay vs. generative construction) yield distinct phenomenological qualities.

Beyond clock and calendar time, temporal cognition also involves navigating ordered sequences. Whether remembering a precise chain of past events or anticipating the likely steps of a future scenario, the ability to represent temporal order is essential for integrating events into coherent narratives and effective plans (Friedman, 1993; Zacks et al., 2007; Baldassano et al., 2017). This connection between cultural mappings of time's arrow and the cognitive mechanics of MTT (Núñez and Sweetser, 2006) highlights how metaphor and memory work together to shape our experience of time.

### 3.3 Grammatical structure and attentional focus

Linguistic influence extends beyond metaphor to the level of grammatical structure, which can direct a speaker's attention to different aspects of an event as it unfolds. Fausey and Boroditsky (2011) investigated how English and Spanish speakers describe and remember intentional vs. accidental events. For intentional acts, both groups used similar, agentive language (e.g., he broke the vase). However, for accidental acts, English speakers still tended to use agentive phrasing, whereas Spanish speakers often used a non-agentive construction common in their language (*se rompió el florero*, literally the vase broke itself) (Fausey and Boroditsky, 2011). This linguistic difference was mirrored in a non-linguistic memory test. Both groups remembered the agents of intentional acts equally well. However, English speakers had significantly better memory for the agents of accidental events than Spanish speakers did (Fausey and Boroditsky, 2011). This suggests that the grammatical patterns of one's language tune attention during event encoding, with lasting consequences for what is remembered (Lucy, 1992).

Cross-linguistic research on grammatical aspect provides evidence that linguistic systems can influence how speakers mentally represent the temporal unfolding of events, a process central to MTT. In experimental studies, English speakers, whose language frequently encodes progressive aspect, focus on the ongoing dynamics of actions, while German speakers, whose language typically leaves such distinctions to context, are more inclined to highlight completion or outcomes (von Stutterheim and Nuse, 2003; Athanasopoulos and Bylund, 2013). These systematic differences in narrative and visual scene description suggest that habitual patterns of language use can influence perceptual encoding, directing attention toward particular temporal features of experience. While earlier in this section we illustrated this distinction with tense–aspect forms in English and German, here the focus is on empirical evidence from discourse production and event segmentation tasks. Athanasopoulos et al. (2015) demonstrated that such differences are not confined to language use: when describing or recalling video clips, English speakers preferentially attended to the intermediate stages of an action, whereas German speakers more often foregrounded its endpoint. This divergence persisted even in non-verbal memory tasks, supporting the view that repeated engagement with a language's grammatical structures can foster enduring attentional biases in event perception.

In a different domain, Chen (2013) explored the relationship between grammar and economic behavior. He classified languages based on whether they have a strong future-time reference (FTR), requiring a distinct grammatical form for future events (like English “will save”), or a weak FTR, where the present tense can be used to refer to the future (like German *morgen spare ich*, tomorrow I save). He found that speakers of weak-FTR languages, which grammatically group the future with the present, exhibit more future-oriented behavior: they have higher savings rates, are less likely to smoke, and accumulate more retirement wealth (Chen, 2013). The proposed mechanism is that if language makes the future seem more immediate, it is discounted less heavily in decision-making.

### 3.4 Cultural constraints on temporal concepts

Some cultural systems for time diverge significantly from the linear, quantified model common in the West. In many traditional African societies, time is understood in terms of the events that occur within it, as opposed to being treated as an abstract commodity to be saved or spent (Mbiti, 1969). Time is reckoned by natural cycles (i.e. planting seasons, harvests, the position of the sun, etc.) and social gatherings. This event-based orientation fosters a two-dimensional view of time, with a long, knowable past (*Zamani*) and a changing present (*Sisa*), but a very limited or non-existent distant future (Mbiti, 1969). The future is only relevant as an extension of the present. This polychronic, relational approach prioritizes social connection over strict punctuality, a cultural value that can lead to misunderstandings in cross-cultural interactions (Babalola and Alokan, 2013).

This general model, however, masks considerable diversity. The Yoruba of Nigeria, for instance, do possess a three-dimensional view of time, including a concept of the future that extends into the afterlife (Fayemi, 2016). Their time is event-based and measured in relation to ecological and celestial markers (e.g., the crowing of the cock, the length of a shadow) as well as the stages of human life (infancy, youth, old age). Similarly, the Igbo people traditionally used a cyclical calendar (Ị*gụ Arọ*) based on the moon and a 4-day week determined by market days associated with different spirits (Ossai, 2016). For both cultures, time is conceived as a qualitative phenomenon embedded in the rhythm of social, spiritual, and ecological life. Beyond Africa, other cultures diverge even further from the linear, metric model.

The case of the Pirahã people of the Amazon, as documented by Everett (2013), presents an even more radical example. The Pirahã language is reported to lack grammatical tense, recursion, and words for both numbers and specific colors (Everett, 2013). Their cultural focus is on immediate, directly verifiable experience, and they reportedly show little concern for the distant past or future. While these claims are the subject of ongoing linguistic debate, the Pirahã case challenges the assumption that complex, abstract temporal structuring is a universal feature of human cognition. It suggests that cultural values can place constraints on the development and expression of the linguistic tools for time, potentially limiting the scope of MTT itself.

Consistent with these cultural biases, many findings across species show that timing is often relative rather than metric (Gibbon, 1977; Gibbon et al., 1984; Buhusi and Meck, 2005). In temporal bisection tasks, rats and pigeons learn a short and a long standard and then classify probe durations as “short” or “long,” with the psychometric midpoint typically near the geometric mean of the standards (Church and Deluty, 1977; Gibbon, 1977; Gibbon et al., 1984). Changing the anchor set shifts this midpoint even when the physical probe duration is unchanged, indicating ratio-based, context-normalized comparison rather than readout from a fixed millisecond scale (Gibbon et al., 1984; Wearden and Lejeune, 2008). Humans show the same bisection pattern—geometric-mean midpoints and anchor-induced shifts—under closely matched procedures (Wearden and Lejeune, 2008; Lejeune and Wearden, 2009). In the peak-interval procedure, response rates from rats and pigeons concentrate around the trained interval and the spread of responding increases proportionally with interval length, expressing the scalar property that variability grows with duration and again pointing to relative coding (Matell and Meck, 2004; Buhusi and Meck, 2005). Related central-tendency and memory-mixing effects pull judgments toward the context average across trials in both humans and rats, further demonstrating comparison against recent temporal context rather than absolute time (Crystal, 1999; Lejeune and Wearden, 2009). These patterns support the claim that, in many timing tasks, a context-normalized comparison suffices without a fixed internal metric (Gibbon, 1977; Buhusi and Meck, 2005).

This possibility is consistent with comparative research on interval timing. Classic experiments in rats show that when trained to discriminate between short and long durations, performance is governed by the ratio between the two rather than their absolute difference (Church and Deluty, 1977; Gibbon, 1977). As a result, their psychophysical responses are centered on the geometric mean of the intervals, indicating a proportion-based encoding of time. Similar scalar, ratio-based timing patterns have been observed across pigeons, monkeys, and humans in non-metric timing tasks (Wearden and Lejeune, 2008).

Anthropological evidence suggests that this relative, proportion-based structuring is not unique to non-human animals. In many small-scale, non-literate human societies, temporal reckoning is embedded in ecological and social events (seasonal changes, crop cycles, ritual sequences) rather than in fixed, abstract units (Gell, 1992; Zerubavel, 1985). These event-based systems often employ cyclic or situational chronologies and standardizing proportional cues (“after the rains”, “half a day's walk”). In these contexts, time is experienced and represented in relative terms, echoing the scalar properties seen in animal timing.

Taken together, these findings suggest that the capacity for mental time travel may operate over fundamentally different temporal frameworks depending on the cultural and ecological context—ranging from absolute, metric chronologies to relative, event-based sequences. Where the latter dominates, the forward projection of events may be less about estimating precise durations than about navigating proportionate relationships between known temporal landmarks, a style of temporal cognition with deep evolutionary roots.

In Section 4, we now shift from these community-level patterns to examining the comparative question of how related capacities appear across species and contexts, and what ecological demands shape them.

## 4 Evolutionary perspectives: the origins of mental time travel

To understand the human capacity for MTT, one should consider evolutionary context: by examining the cognitive abilities of non-human animals, we can identify which components of MTT might be shared through common ancestry, which may have evolved convergently in response to similar ecological pressures, which ones may have evolved independently due to different ecological pressures, and which may be uniquely human. The comparative approach provides insights into the origins and adaptive functions of temporal cognition. A central tenet of this perspective is that any memory system's ultimate adaptive advantage lies in its ability to inform future behavior, suggesting that memory evolved with the future in mind (Schacter et al., 2007; Dudai and Carruthers, 2005; Dudai, 2009; Clayton and Wilkins, 2018).

Across taxa, experiments reveal flexible recombination rather than fixed routines, which supports a graded, episodic-like construction of mental time travel. In corvids, scrub-jays bind information about what happened where and when, and update their memories of their caches in terms of perishability, social risk, and recency: in the classic perishability design, most Degrade birds switched from worm sites at short delays to peanut sites after long delays while Replenish birds continued to search worm sites at both delays (about 6/8 in each group showed the predicted first-search pattern), and mean items cached were comparable across test trials (≈7.3 vs. ≈7.8), indicating that shifts reflected remembered structure rather than caching-rate differences (Clayton and Dickinson, 1998; Clayton et al., 2001b). Social context produces equally systematic changes: after being watched, birds re-cached about two items on average vs. close to none after private caching, and the proportion re-cached rose from about 6–8% in private to about 28% at Cambridge or about 44% at Davis (Emery and Clayton, 2001; Dally et al., 2006). In rodents, temporal-order and what–where–when tasks with transfer and devaluation controls argue against simple cached values. In the odor-sequence task, control rats were accurate on order judgements across lags of one, two, and three intervening items, averaging about seventy-one to eighty percent correct (≈71.4% at lag 1, ≈79.3% at lag 2, ≈80.0% at lag 3). Rats with hippocampal lesions hovered near chance at each lag, scoring about fifty-eight to sixty-seven percent (≈58.1%, ≈59.3%, ≈67.1%), while recognition accuracy for recent occurrence remained high in both groups during training blocks (controls ≈70.9% → 82.3% → 89.6%; lesioned ≈74.3% → 81.7% → 91.4%), isolating the deficit to temporal order rather than odor recognition *per se* (Fortin et al., 2002). In a radial-maze what–where–when design, rats revisited the chocolate location much more often after a long retention interval than after a short one, rising from about one quarter of revisits to roughly one half (proportion of chocolate revisits ≈0.25 short vs. ≈0.54 long), while revisits to the non-chocolate location stayed high across intervals (≈0.96 short vs. ≈0.90 long). When chocolate was devalued with lithium chloride, revisits to the chocolate site dropped significantly, showing outcome sensitivity rather than a fixed rule (Babb and Crystal, 2005). Converging human and non-human data therefore favor a hippocampal–cortical system that supports constructive recombination with content-dependent specializations rather than species-unique, dedicated future-only modules (Schacter and Addis, 2007; Allen and Fortin, 2013; Templer and Hampton, 2013).

Given the deep phylogenetic split and distinct neural architectures, functional similarities between cephalopods and vertebrates are most parsimoniously interpreted as convergent responses to similar ecological problems, for example irregular resource cycles, spatially structured foraging, and social competition that benefit from flexible reconstruction and future use of event information (Emery and Clayton, 2004; Seed et al., 2009; Amodio et al., 2019; Schnell and Clayton, 2021). Convergence here refers to alignment at the level of function and strategy with differences in implementation, which fits evidence that octopus and cuttlefish achieve flexible, context-dependent choices using neural substrates and life histories unlike those of birds and mammals, yet show task outcomes that match criteria for episodic-like construction (Jozet-Alves et al., 2013; Billard et al., 2020a,b; Schnell et al., 2021c). This interpretation does not require that intervening lineages lack the capacity. It predicts a mosaic distribution in which elements of episodic-like use appear across taxa when ecological pressures make them useful, and it allows that some core computations could be broadly conserved while being assembled into different systems across clades (Corballis, 2013). The comparative claim is therefore graded: similar task performance under stringent criteria, coupled with divergent circuitry and matching ecological demands, supports convergence, while leaving open limited homology at the level of general mnemonic operations.

Tool limitations do not preclude tests of foresight. As tool-free alternatives to Tulving's Spoon Test—i.e., planning for a future need without prior associative training (Tulving, 2005; Wilkins and Clayton, 2019)—ecologically grounded “deferral without manipulation” paradigms demonstrate prospective use of remembered structure across taxa. In corvids, scrub-jays seed tomorrow's deprived compartment and re-cache after being watched, with adjustments contingent on who observed and what was cached (Raby et al., 2007; Emery and Clayton, 2001; Dally et al., 2006). In rodents, temporal-order and what–where–when tasks tie payoffs to delayed, context-specific retrieval and include transfer and devaluation controls that discount simple cached values (Fortin et al., 2002; Babb and Crystal, 2005). In cephalopods, cuttlefish modulate foraging as availability cycles and wait for higher-value prey when delay improves outcome quality, revealing graded self-control without any requirement to handle tools (Jozet-Alves et al., 2013; Billard et al., 2020b; Schnell et al., 2021a).

### 4.1 Episodic-like memory in non-human animals

The comparative study of memory advanced through research on food-caching corvids. In their seminal study, Clayton and Dickinson (1998) tested whether California scrub-jays (*Aphelocoma californica*) could remember the ‘what, where, and when' of previous caching events by allowing the birds to hide perishable wax worms and non-perishable peanuts, and recover their caches after a short delay of 4 h or a long delay of 124 h. The birds had been hand-raised so that their experience of whether or not worms perish could be manipulated. The critical manipulation for the Degrade group was the retention interval between caching and recovery: after a short delay of 4 h, birds preferentially searched for worms, whereas after a long delay of 124 h (4 h and 5 days later to control for circadian rhythms), they avoided worm caches and searched for peanuts. By contrast, the Replenish group only ever experienced the worms fresh and therefore selectively searched for worms after both retention intervals. On test trials all caches were removed before recovery so that the birds could not rely on cues emanating directly from the food, such as smell or color of the sand, and therefore had to rely on memory for what they had hidden, where, and when. Subsequent studies (Clayton and Dickinson, 1999) established that the jays did not rely on relative familiarity of the caching trays by allowing the birds to cache worms on one side of the tray 120 h before or after they had cached the peanuts, so that the retention intervals of 4 and 124 h between caching and recovery remained the same and the only way to solve the task was through memory recall. In the absence of any agreed behavioral tests of phenomenological consciousness (Griffiths et al., 1999), both autonoetic and chronosthetic, Clayton and Dickinson (1998) coined the term episodic-like memory to refer to this ability of the jays to recall the ‘what, where, and when' of past events without any reference to these phenomenological aspects of mental time travel.

Clayton et al. (2001b) argued that episodic-like memory should be defined as an integrated what–where–when representation within a declarative system, rather than as separate capacities for space, time, and event. They proposed three operational criteria: content, the memory specifies what occurred, where, and when; structure, these elements are bound into a single representation; and flexibility, the information can be updated after encoding and applied to novel problems in a manner characteristic of declarative, not procedural, memory.

Subsequent experiments demonstrated that WWW memory in scrub-jays is not restricted to a fixed decay rule for wax worms. Clayton et al. (2001b, 2003) trained birds with novel perishable items, mealworms and crickets, characterized by distinct degradation rates. Mealworms degraded within 1 day, whereas crickets remained edible after 1 day and spoiled only after 4 days. Jays adjusted retrieval behavior according to these learned perishability rates, indicating that perishability information is stored in a food-specific manner. Clayton et al. (2003) suggested that the learned information about perishability rates was akin to semantic knowledge, whereas the memory for where they had hidden the specific food items and how long ago was akin to remembering episodic information about specific past events.

The Clayton et al. (2003) experiment provided a critical test of this declarative flexibility. In phase one, birds received a test trial with a novel retention interval of 3 days. All jays preferentially searched sites where they had previously cached crickets, consistent with the expectation that crickets might still be fresh. In phase two, an interleaved caching procedure provided new information: for the Consistent group, crickets were indeed fresh after 3 days; for the Inconsistent group, they had degraded. On day six, during a probe trial for caches made in a third tray, birds in the Consistent group searched where they had cached crickets, whereas birds in the Inconsistent group selectively searched in sites where they had cached peanuts, showing retroactive updating of a past event representation with knowledge acquired long after the original episode (Clayton et al., 2003). Additional work extended these findings to positive temporal changes in food value when food ripens rather than degrades. For example, de Kort et al. (2005) demonstrated that scrub-jays could track the ripening of initially unpalatable food items over multi-day intervals and adjust recovery to coincide with peak palatability, ruling out directed forgetting and reinforcing sensitivity to temporal dynamics in both spoilage and ripening contexts.

Collectively, this body of work indicates a flexible, context-dependent memory system capable of integrating temporal information about event content, location, and timing, and of updating past representations in light of subsequent experience. Since then, many other animals have been shown to possess episodic-like what–where–when memory, although few studies probe declarative flexibility with the same controls for differing perishability and ripening rates (Jelbert and Clayton, 2017; Worsfold et al., 2025).

### 4.2 Future planning and foresight

If episodic-like memory supports prospective use, animals that show integrated what–where–when representations should also act for a future need that differs from their current state. This criterion is captured in the Bischof–Köhler test, which asks whether behavior is organized around tomorrow's contingencies rather than today's motivation (Suddendorf and Corballis, 1997; Davies and Clayton, 2024). A trial-unique breakfast-room design shows such prospection in scrub-jays. Birds first experienced two bedrooms that differed only in what would happen the next morning, one reliably provided breakfast, the other did not. During this phase, only powdered food was available, which blocked any caching reinforcement. On a single, unexpected evening they received whole items that could be cached. If birds used remembered contingencies to meet a future need, they should seed the room that would lack breakfast. They did: the night before, they cached substantially more in the no-breakfast room than in the breakfast room (about 16.3 items on average vs. about 5.4; *n* = 8; t7 = 3.01, *p* = 0.02), despite being sated and receiving no outcome-based feedback for correct placement (Raby et al., 2007).

Social contexts reveal complementary foresight. Scrub-jays that have prior pilfering experience later move caches they made under observation when they get a chance to revisit in private, effectively invalidating the observer's knowledge. This re-caching depends on episode-specific information about who watched and what was cached, consistent with projecting a competitor's likely future theft based on remembered observation history. Birds moved about two items on average after being watched vs. close to none after private caching, and the proportion re-cached rose from roughly 6–8% in private to about 28–44% when caches had been observed (Emery and Clayton, 2001; Dally et al., 2006).

Comparable deferral-without-manipulation approaches extend beyond corvids. In rodents, temporal-order and what–where–when paradigms tie payoffs to delayed, context-contingent retrieval and include transfer and devaluation controls that discount simple cached values (Fortin et al., 2002; Babb and Crystal, 2005). In cephalopods, cuttlefish modulate foraging as availability cycles and wait for higher-value prey when delay improves outcome quality, which indicates decisions guided by expected future states rather than immediate reward (Jozet-Alves et al., 2013; Billard et al., 2020b). In a delay-of-gratification task, individuals tolerated waiting times that varied widely, from roughly 50 s to about 130 s, and the capacity to wait was positively related to reversal-learning performance (correlation about *r* = 0.77), indicating graded self-control rather than an all-or-none effect (Schnell et al., 2021a). Age did not depress episodic-like performance: out of six mature and six old cuttlefish, five of six and six of six, respectively, met the criterion over three consecutive test days, and the proportion of correct trials did not differ significantly between age groups (*p* ≈ 0.179) (Schnell et al., 2021b).

### 4.3 Independent evolution of cognition

The presence of advanced cognitive abilities in corvids, whose lineage diverged from that of mammals approximately 320 million years ago, provides robust evidence that complex cognition can evolve independently in phylogenetically distant vertebrate taxa. Comparative analyses indicate that corvids and great apes exhibit convergent evolution in multiple cognitive domains, including episodic-like memory, flexible future planning, and complex social cognition (Emery and Clayton, 2004). These similarities are generally attributed to parallel selective pressures, such as the demands of exploiting temporally and spatially variable food resources and managing intricate social relationships.

Cephalopods, and in particular cuttlefish, provide an even more phylogenetically distant comparison. Their lineage diverged from vertebrates over 550 million years ago, and their central nervous system is organized according to a fundamentally different architectural plan, lacking a hippocampal formation. Nevertheless, cuttlefish possess episodic-like memory. In cross-modal source-memory designs, the same cue signals different outcomes in distinct learning contexts, visually or spatially marked with counterbalanced reinforcement histories; after a delay, animals are tested with perceptually identical cues, so successful choice requires retrieving where and under which conditions the contingency was learned rather than relying on immediate stimulus–response strength. Performance is reliably above chance: all cuttlefish chose the correct scene panel after a 1-h delay (exact binomial *p* ≈ 0.0039; 95% CI ≈ 0.66–1.00), and accuracy after 3 h remained above chance (*p* ≈ 0.035; 95% CI ≈ 0.46–0.94). Training to criterion was graded, three of ten individuals reached criterion on day one, rising to nine of ten by day six, indicating group-level generalization rather than a single exceptional performer (Billard et al., 2020a,b; Jozet-Alves et al., 2013).

Although these findings are consistent with the independent evolution of episodic-like and future-oriented cognition, the case for direct convergent evolution with corvids and apes is less likely in cephalopods. Convergent evolution presupposes similar selective regimes. For corvids and apes, both ecological complexity and social complexity are implicated in the emergence of advanced cognition. Cuttlefish, by contrast, are largely asocial, lacking the selective pressures associated with group living that are thought to facilitate the evolution of theory of mind, recursive perspective-taking, and a socially embedded concept of self (Amodio et al., 2019; Schnell et al., 2021c; Schnell and Clayton, 2021). Their cognitive abilities may therefore be primarily adaptations to solitary foraging in dynamic environments, coupled with the demands of flexible camouflage and predator avoidance. This distinction implies that while certain cognitive functions, such as integrating episodic information to guide future behavior, may be phylogenetically widespread, the full suite of socio-cognitive specializations observed in corvids and apes may be contingent upon social selective pressures.

In humans, MTT appears exceptional in temporal scale, flexibility, and integration with other cognitive systems. Whereas, scrub-jays may plan for the next day's feeding, humans routinely simulate events decades in the future, embed these simulations in counterfactual and hypothetical frameworks, and incorporate them into moral reasoning and cultural narratives. The Spoon Test (Tulving, 2005; Wilkins and Clayton, 2019) operationalises a stringent criterion for MTT, the capacity to select a tool in the present for use in a novel problem-solving context in the future, without prior associative training. Evidence from New Caledonian crows indicates that at least some non-human species can meet aspects of this criterion: crows flexibly select tools for later use in a different context and adapt choices when anticipated future conditions change, with subsequent work addressing methodological critiques and reinforcing interpretation as future-oriented planning rather than simple associative rules (Boeckle et al., 2020, 2021). Shared MTT via language may represent the defining elaboration of this capacity in humans. By encoding and communicating mental simulations, individuals can transfer both concrete experiences and abstract, counterfactual, or purely imagined scenarios to others, enabling intergenerational transmission of knowledge, cooperative design of future actions, and the maintenance of complex social institutions (Garcia-Pelegrin et al., 2021). In evolutionary terms, the transformation of an individually adaptive cognitive process into a collectively shared symbolic system likely amplified the selective advantage of MTT, producing a qualitative discontinuity between human temporal cognition and that of other taxa.

## 5 Modulators and vulnerabilities of temporal memory accuracy

Constructive operations at encoding and reconstruction at retrieval both support foresight and introduce characteristic vulnerabilities. We focus on two influences with practical weight for temporal accuracy: the malleability of recollection under misinformation and the effects of biological timing, including circadian cycles and sleep-dependent consolidation (Loftus and Palmer, 1974; Loftus, 2005; Gerstner and Yin, 2010). Two concise orienting frameworks, Schacter's seven sins of memory and Clayton and Wilkins' seven myths of memory, illustrate common error patterns and misconceptions (Schacter, 1999, 2001, 2022; Clayton and Wilkins, 2018); the scope spans neuropsychology, experimental cognitive psychology, comparative cognition, and biological timing (interval timing, circadian rhythms, and sleep-dependent consolidation).

Neuropsychological data clarify functional links between remembering and future thinking. Patient K.C., following bilateral medial temporal lobe damage, retained temporal knowledge for when events occurred (e.g., clock or calendar placement) while lacking autonoetic re-experience of past episodes and the capacity to construct detailed personal futures, revealing a dissociation between temporal semantics and self-projected episodic simulation (Tulving, 2002a,b; Rosenbaum et al., 2005). Present-oriented biases further shape retrospection and prospection: current beliefs and affect systematically influence remembered pasts and predicted futures, consistent with findings on consistency bias and affective forecasting (Wilson and Gilbert, 2005; Gilbert and Wilson, 2007).

Extreme mnemonic phenotypes demonstrate that accessibility and accuracy dissociate. Individuals with Highly Superior Autobiographical Memory (HSAM) show dense, date-specific recall yet remain vulnerable to misinformation and false memories (Parker et al., 2006; LePort et al., 2012; Patihis et al., 2013). These considerations motivate an adaptive account in which constructive and reconstructive operations serve prediction and planning, with errors emerging as trade-offs of that design (Schacter and Addis, 2007; Schacter, 2012). Subsequent subsections examine mechanisms and modulators in detail, including the misinformation effect (Section 5.2) and influences of biological timing, including circadian cycles and sleep-dependent consolidation, with complementary systems-level evidence summarized in Section 2. There we review hippocampal–neocortical dialogue across NREM and REM, reactivation and replay phenomena in rodents and songbirds, and related human findings that bear on constructive simulation (Ji and Wilson, 2007; Dave and Margoliash, 2000; Diba and Buzsáki, 2007; Rasch and Born, 2013). Here in Section 5, we focus on consequences for temporal accuracy, namely when circadian phase and sleep stage improve or degrade order memory, duration judgments, and prospective remembering, and how aligning encoding, retrieval, and decision windows to biological rhythms yields better outcomes (Plihal and Born, 1997; Gerstner and Yin, 2010; Roenneberg et al., 2019).

### 5.1 Brief orienting frameworks

Two concise orienting frameworks summarize failure modes and misconceptions that matter for temporal accuracy and for how people use mind time in everyday planning. Schacter's seven sins of memory describe patterned errors that arise from normal operation rather than breakdown (Schacter, 2001, 2022). Clayton and Wilkins' seven myths of memory target widespread but incorrect beliefs about how memory works, including beliefs about temporal precision and the status of eyewitness reports (Clayton and Wilkins, 2018). We present both here to anchor later references rather than to rehearse full debates.

Schacter's sins organize common error patterns into omissions and commissions that are directly relevant to timing, ordering, and foresight. Transience is loss with delay that shifts remembered durations and the order of events, which matters for prospective plans made long after encoding. Absent-mindedness reflects lapses at encoding or retrieval that impair time-based intentions when monitoring demands are high. Blocking is temporary retrieval failure that removes key temporal landmarks even when the event can be recognized. Misattribution is assignment of a remembered detail to the wrong source, which is critical for dating events and for separating what happened then from what was learned later. Suggestibility is incorporation of misleading information that can distort temporal sequence and duration after exposure to post-event cues (Loftus and Palmer, 1974; Loftus, 2005; Mitchell and Johnson, 2009). Bias is systematic reshaping by current beliefs and goals that can pull remembered timelines toward present expectations. Persistence is intrusive remembering that can overweight salient temporal anchors and crowd out more diagnostic cues. These patterns explain why temporal memory is not uniformly precise, and why design choices that reduce misleading cues or lower monitoring load can improve prospective performance in natural settings.

Clayton and Wilkins' myths clarify what memory does not do and why certain interventions fail. The video-camera myth is the belief that memory preserves a complete record, when in practice reconstruction fills temporal gaps from gist and context. The confidence-equals-accuracy myth is the belief that certainty guarantees correctness, although high confidence can coexist with demonstrable error, including in HSAM and amnesic cases (Parker et al., 2006; LePort et al., 2012; Patihis et al., 2013; Rosenbaum et al., 2005). The emotion-protects-detail myth is the belief that arousal secures accurate chronology, when elevated arousal can narrow attention and systematically distort perceived duration (Droit-Volet and Meck, 2007; Droit-Volet and Gil, 2009). The repetition-guarantees-truth myth is the belief that rehearsal makes memories more accurate, when repetition can increase fluency and coherence without guaranteeing veridical order. The recovery-techniques myth is the belief that special procedures reliably restore lost memories, when such methods can increase suggestibility and source confusions (Zaragoza and Lane, 1994). The eyewitness-is-definitive myth is the belief that testimony gives verbatim access to the past, when post-event language and questioning shape both content and temporal assignment (Loftus and Palmer, 1974; Fausey and Boroditsky, 2011). The single-system myth is the belief that memory is unitary, when different operations contribute to construction, monitoring, and temporal control (Simons and Spiers, 2003; Schacter and Addis, 2007). We return to these points in Section 5.2, where misinformation, source confusion, and dissociations between confidence and accuracy are examined with the classic paradigms and controls.

In this manner, the sins and myths complement the rest of the review. In Section 2, constructive operations that enable simulation also create avenues for misattribution and bias, which later interact with misinformation and biological timing in Section 5. In Section 3, modest linguistic and cultural influences shift defaults for temporal reference and can interact with suggestibility and bias without determining individual outcomes. In Section 4, comparative evidence is evaluated with attention to design features that guard against misattribution and suggestibility. In Section 6, externalized time and shared artifacts counter specific sins by stabilizing order and duration outside memory while still respecting the event-relative structure people use in mind time.

### 5.2 The misinformation effect and memory malleability

The sin of suggestibility is powerfully demonstrated by the misinformation effect, a phenomenon extensively documented by Loftus and colleagues. Research has shown that exposure to misleading information after an event can alter an individual's memory of that event (Loftus, 2005). In a classic paradigm, participants witness a complex event (e.g., a simulated car accident). Later, some participants are exposed to misinformation (e.g., a misleading question that refers to a “yield sign” when the original scene contained a “stop sign”). When subsequently tested, these participants often incorporate the misinformation into their memory, confidently reporting that they saw the yield sign (Loftus and Palmer, 1974).

This effect is strong and can be used to plant memories of entirely false events, such as being lost in a shopping mall as a child or witnessing demonic possession (Loftus, 2005). Several mechanisms have been proposed to explain this phenomenon. The memory replacement hypothesis suggests that the misleading information overwrites and destroys the original memory trace. Alternatively, the memory coexistence hypothesis posits that both the original and the misleading information remain in memory, but the misinformation is more accessible and therefore more likely to be retrieved. A third account focuses on source misattribution, arguing that individuals remember both pieces of information but incorrectly attribute the source of the misinformation to the original event (Zaragoza and Lane, 1994).

Highly Superior Autobiographical Memory (HSAM) provides a direct test of whether exceptionally detailed autobiographical access protects against suggestibility. The first case report, AJ (Jill Price), documented striking, date-specific recall for personal and public events (Parker et al., 2006). Group studies subsequently confirmed unusually dense autobiographical access in HSAM (LePort et al., 2012). HSAM does not confer immunity to misinformation: HSAM individuals remain susceptible to false memories in list-learning and misinformation paradigms (Patihis et al., 2013). These findings indicate that high accessibility does not guarantee veracity and that susceptibility to suggestion arises from the reconstructive nature of remembering. This pattern is consistent with the Spoon Test analysis, which emphasizes memory's organization around prospective utility rather than verbatim archival accuracy (Wilkins and Clayton, 2019).

Regardless of the precise mechanism, the misinformation effect provides compelling evidence for the reconstructive nature of memory. The same neural and cognitive mechanisms that allow for the flexible recombination of details to simulate the future (as described in Section 2) also leave memory susceptible to incorporating externally supplied details into its reconstructions of the past. The brain does not appear to have a foolproof “tag” for distinguishing internally generated simulations from recollections of external events, making memory malleable (Conway, 2009).

### 5.3 Interval timing and circadian rhythms

Memory formation and consolidation are not static processes; they are dynamically modulated by internal biological clocks operating on multiple timescales. These temporal control systems matter because when information is encoded or retrieved, and the precision of temporal markers available at those moments, affects accessibility, interference, and updating, i.e., several error patterns often discussed under the “seven sins” framework (e.g., transience, absent-mindedness). As a transition from externally supplied distortions (Section 5.2), this section focuses on endogenous timing mechanisms that gate encoding and retrieval. Following Friedman, memory for when an event occurred can be supported by multiple cues—distance (how long ago), location (calendar/clock), and relative order—providing several ways to “do time” without invoking autonoetic mental time travel (Friedman, 1993, 2004). These mechanisms are dissociable from episodic MTT: they can operate in the absence of autonoesis and often run concurrently with, rather than constituting, episodic simulation (Hoerl and McCormack, 2019).

#### 5.3.1 Interval timing

While MTT concerns the autobiographical scale, day-to-day functioning depends on a finer-grained temporal sense: interval timing, the perception of durations in the seconds-to-minutes range. This capacity supports motor control, speech perception, and decision-making (Buhusi and Meck, 2005). Interval timing and MTT are dissociable processes: success in one neither implies nor requires success in the other, although both can inform how experiences are encoded and later reconstructed (see Friedman's distance/location/order markers above; Hoerl and McCormack, 2019).

The dominant psychological account is Scalar Expectancy Theory (SET), or the “internal clock” model (Gibbon et al., 1984). SET posits (1) a pacemaker emitting pulses, (2) an accumulator that counts pulses when attention is directed to time, and (3) a comparator that matches the count to reference memory. SET explains the scalar property: variability in time estimates scales proportionally with the interval (Lejeune and Wearden, 2009). A canonical demonstration is temporal bisection in rodents: after training on “short” (e.g., 3 s) vs. “long” (e.g., 12 s) signals, the point of subjective equality falls at the geometric rather than arithmetic mean (≈6 s here), indicating ratio-based timing (Church and Deluty, 1977). This ratio signature suggests an efficient coding scheme for duration and motivates cross-cultural hypotheses: in populations with limited exact number lexicons (e.g., “one–two–three–few–many”), relative/ratiometric strategies may be favored, paralleling classic results in numerical cognition (Gordon, 2004; Pica et al., 2004).

Neurobiologically, interval timing is supported by a distributed network. The Striatal Beat Frequency (SBF) model proposes that populations of cortical oscillators are reset at interval onset; the striatum acts as a coincidence detector that learns patterns predictive of reinforcent. Dopamine modulates the effective clock speed such that increased dopaminergic tone yields overestimation of duration (Matell and Meck, 2004; Meck and Malapani, 2004). This cortico-striatal timing system is distinct from, but interacts with, hippocampal circuits implicated in episodic memory, providing multiple, interacting mechanisms for processing time at different scales and for shaping when, and how, memory traces are stabilized or accessed. This returns us to the overarching theme: timing control (seconds to minutes) sets windows for encoding and retrieval, thereby constraining what is later reconstructed and potentially biasing which details persist or fade.

#### 5.3.2 Circadian modulation and sleep

Endogenous timing systems shape when traces are encoded, reactivated, and integrated, biasing which details persist, generalize, or fade. On a much longer timescale, memory is governed by the circadian system, a network of endogenous oscillators synchronized by environmental cues (zeitgebers) like the light–dark cycle, which governs nearly all physiological processes on a roughly 24-h cycle (Gerstner and Yin, 2010). In humans and other mammals, the master pacemaker is the suprachiasmatic nucleus (SCN) of the hypothalamus, which coordinates peripheral clocks throughout the brain and body (Smale et al., 2003). In birds, circadian organization is distributed across an avian SCN together with oscillators in the pineal gland and retina, jointly entraining daily sleep–wake and learning-relevant behaviors (Cassone, 2014). In cephalopods, central circadian architecture remains less well characterized; however, emerging work on sleep-like states suggests conserved or convergent organizational principles relevant to memory processing (Frank et al., 2012; de Souza Medeiros et al., 2021; Pophale et al., 2023).

The hippocampus exhibits strong circadian rhythmicity in its gene expression and cellular activity (Eckel-Mahan et al., 2008). Molecular cascades essential for synaptic plasticity and long-term memory (LTM) formation, such as the cyclic AMP-responsive element-binding protein (CREB) pathway, oscillate over 24 h (Gerstner and Yin, 2010). This has direct consequences for learning and memory. The time of day at which information is learned can significantly impact the strength and persistence of the resulting memory, a phenomenon known as the “time-of-day effect” (Chaudhury and Colwell, 2002).

Sleep is actively involved in memory consolidation. Sleep involves intense neural activity during which memories are stabilized, integrated, and reorganized, contrasting with the traditional view of it as a passive state of rest (Rasch and Born, 2013). The active systems consolidation model proposes that during sleep, particularly during non-REM (NREM) slow-wave sleep (SWS), memories are reactivated in the hippocampus. This reactivation, marked by sharp-wave ripples, occurs in coordination with slow oscillations in the neocortex and thalamo-cortical sleep spindles. This “hippocampal–neocortical dialogue” is thought to mediate the gradual transfer of memories from their initial dependence on the hippocampus to more permanent storage in distributed neocortical networks (Ji and Wilson, 2007; Rauchs et al., 2008). Different sleep stages appear to support different types of memory; for instance, SWS is particularly important for consolidating declarative, hippocampus-dependent memories, while REM sleep may be more critical for consolidating emotional memories and procedural skills (Plihal and Born, 1997). Sleep strengthens and transforms memories, extracting the “gist” or general meaning from specific experiences and integrating them with existing knowledge schemas (Stickgold and Walker, 2013).

In songbirds, replay-like activity during sleep is well documented in the zebra finch (*Taeniopygia guttata*), where premotor song regions exhibit patterns that mirror daytime singing, consistent with off-line rehearsal in vocal learning (Dave and Margoliash, 2000), providing a comparative perspective. Evidence is strongest in zebra finches; converging observations across other oscine songbirds are more limited at present. For corvids, direct sleep-stage–specific consolidation evidence remains sparse; while systems-level studies are advancing, replay during identified sleep stages has yet to be established (cf. Nieder, 2020). In cephalopods, alternating quiet and active sleep states have been described, with the active state showing wake-like neural signatures and rapid skin patterning, suggestive of functional specialization akin to vertebrate REM (Frank et al., 2012; de Souza Medeiros et al., 2021; Pophale et al., 2023). The patterns above show predictable shifts from biological timing and misinformation. Section 6 considers how tools that externalize time can counter those shifts when they respect event relations, and how they can worsen them when they impose rigid schedules that ignore how people mentally structure events.

## 6 Externalizing time

Here we examine how externalized time systems, from clocks and calendars to shared scheduling tools, can support event-relative organization in mind time or create friction when they impose strictly clock-first reasoning. In most of human history and during early development, temporal experience is structured by the rhythm of events rather than represented as an abstract quantity. The passage of time was marked by the rising and setting of the sun, the changing of the seasons, the cycles of hunger and satiety, and the sequence of daily activities (van der Weel, 2011). This “event-time” is concrete and content-rich; it is a qualitative experience where the duration of an activity is defined by the task itself, not by an external, arbitrary unit (Levine, 1997). One finishes when the task is done, not when the clock strikes the hour. The invention and widespread adoption of mechanical clocks, a process that began in European monasteries in the 14th century, introduced a fundamentally different way of conceiving time: as an abstract, uniform, and objective medium that flows independently of the events that occur within it (Zerubavel, 1982). This section examines how these externalized time-keeping technologies have reshaped human temporal cognition, creating a uniquely modern psychological landscape.

### 6.1 Development of temporal cognition and the role of external supports

From the pre-school years, children begin to use temporal vocabulary and simple before/after sequencing, but judgments are initially tied to salient routines and recent experience rather than to abstract temporal metrics (Friedman, 2003; Droit-Volet, 2013). Between roughly ages 4 and 7 there is measurable growth in episodic future thinking and in simple prospective memory, with better performance on event-based tasks where environmental cues are perceptually available at the moment of action (Atance and O'Neill, 2005; Einstein and McDaniel, 2008; Mahy et al., 2014; Shelton and Scullin, 2017). Across the primary school years, improvements in executive control, working memory, and temporal estimation support a shift toward conventional timekeeping; children increasingly map activities to clock and calendar units and begin to plan multi-step sequences that extend beyond the here-and-now (Wearden, 2016; McCormack and Hoerl, 2017; Droit-Volet, 2013). Importantly, developmental change in temporal estimation is also characterized by qualitative shifts in the heuristics used to judge duration, rather than only by a uniform scaling of general cognitive capacity: when comparing equal-length “eventful” and “uneventful” intervals, pre-kindergarten children tend to judge the eventful interval as longer, whereas school-age children and adults tend to judge the uneventful interval as longer, consistent with different age-graded strategies for extracting temporal information (Stojić et al., 2023).

Development in related cognitive domains supports this change. Growth in narrative organization helps children order events causally and temporally, while gains in theory of mind and counterfactual reasoning make it easier to anticipate the informational needs of others and coordinate joint plans that unfold over time (Suddendorf and Corballis, 2007; McCormack and Hoerl, 2017). These capacities interact with time perception: children's temporal bisection and reproduction become more accurate and less context-bound with age, which reduces reliance on salient event markers and enables a more stable use of conventional temporal units for planning and recall (Wearden, 2016; Droit-Volet, 2013). At the same time, the informational basis of duration judgments can reorganize across development. Stojić et al. (2023) interpret their polarized group patterns as a transition from an availability heuristic in pre-kindergarteners (where event density and perceptual change are treated as diagnostic of “more time”) toward a sampling heuristic by school age, alongside increasing reliance on an observer-independent notion of duration in adults (Stojić et al., 2023). This kind of heuristic shift helps explain why “more happening” can feel longer earlier in development, while later judgments may be driven more by how time is sampled, monitored, or reconstructed when events are sparse (Stojić et al., 2023).

External tools play a practical role throughout this progression. Visual schedules, checklists, timers, and shared family or classroom calendars act as memory supports that reduce monitoring demands and stabilize temporal reference, with the strongest benefits when cues are proximal to the target action and embedded in the relevant context (Dismukes, 2012; Mahy et al., 2014; Rummel and Kvavilashvili, 2022). In developmental terms, these supports can scaffold whatever heuristic is currently dominant: for younger children, event-anchored prompts and routine-linked cues may align with duration judgments that are sensitive to eventfulness and availability (Stojić et al., 2023), whereas later the same children increasingly benefit from tools that externalize symbolic conventions (clock faces, timetables, weekdays) as stable coordination media (McCormack and Hoerl, 2017; Wearden, 2016).

As children age, reliance shifts from purely event-bound cues toward symbolic systems such as clock time and weekdays. Even adolescents and adults perform best when tools respect event-relative structure, for example “after homework, pack the bag,” rather than forcing all coordination through abstract clock time alone (McCormack and Hoerl, 2017). These developmental patterns clarify why externalized time-keeping and shared schedules can improve coordination in everyday settings. They also indicate how mismatches between mind time and clock time may be reduced through designs that align cues with intended actions, preserve meaningful relations among events, and allow conventional units to be used without overriding the event structure that people rely on for recall and simulation.

### 6.2 Externalized time vs. mind time

Mumford (1934) argued that “the clock, not the steam-engine, is the key machine of the modern industrial age.” Before the mechanical clock, timekeeping was tied to natural, often irregular cycles. Sundials were useless on cloudy days, and water clocks were imprecise and susceptible to environmental changes. The mechanical clock, with its regular, oscillating escapement, dissociated time from human events and natural rhythms. It created a mathematical, predictable, and divisible time. As Mumford noted, “time-keeping passed into time-serving and time-accounting and time-rationing… one ate, not upon feeling hungry, but when prompted by the clock: one slept, not when one was tired, but when the clock sanctioned it.” This shift represented a cognitive reorientation extending beyond technological change.

The full societal impact of this reorientation was realized with the advent of industrialization and, particularly, railway transportation. As long as each town operated on its own local solar time, coordinating train schedules was a logistical impossibility (Zerubavel, 1982). The need for synchronization drove the establishment of standardized time zones in the 19th century, first by railway companies and later codified by governments. This process imposed an external, artificial grid onto both social life and subjective experience, creating a constant cognitive tension between two competing temporal systems: an internal, subjective, event-driven “mind time” and an external, objective, abstract “clock time” (Bejan, 2019). Mind Time denotes the self's event-based pacing and goal-directed navigation of experience; by contrast, Tulving's “chronesthesia” refers to conscious awareness of temporal relations themselves (e.g., before/after/now, duration). Standardized clock time tends to regularize chronesthetic judgements, yet it often conflicts with mind time's endogenous pacing, forcing subjective temporal flow to entrain to external schedules.

At a cognitive level, the increasing reliance on clock time in adolescence and adulthood can be framed as a representational shift that is analogous to that observed in spatial cognition: a transition from egocentric to allocentric reference frames (Stojić and Nadasdy, 2024). On this account, time perception remains fundamentally event-centered across development, in the sense that events provide the primary units for segmenting experience and for constructing psychological time (Stojić and Nadasdy, 2024). What changes is the dominant perspective used to locate and estimate durations: younger individuals rely more on egocentric, availability-based heuristics anchored to event density and what is readily retrievable, whereas older individuals increasingly adopt allocentric strategies that treat duration as an observer-independent dimension that can be sampled and coordinated against shared conventions (Stojić and Nadasdy, 2024).

This proposed shift is supported by experimental evidence that different age groups apply distinct heuristic rules when judging equal objective durations. In a retrospective comparison of an “eventful” and an “uneventful” 1-min interval, pre-kindergarteners tended to judge the eventful interval as longer, while school-age children and adults tended to judge the uneventful interval as longer (Stojić et al., 2023). Stojić et al. (2023) interpret this reversal as an age-dependent switch from an availability heuristic (“how much they can talk about something,” in which event density drives perceived duration) to a sampling heuristic (“how many times they could sample the flow of absolute time,” in which sparse content affords more “time awareness” moments, or more opportunities to consult external time) (Stojić et al., 2023). In their broader synthesis, Stojić and Nadasdy (2024) further argue that an allocentric perspective on time becomes especially salient later in adolescence, as personal life becomes organized narratively and autobiographical memories are reorganized along a more explicit mental timeline (Stojić and Nadasdy, 2024).

This tension manifests in numerous ways. The common experience of time “flying” during an engaging activity or “dragging” during a boring one is a direct result of the mismatch between these two systems. Subjective duration is heavily influenced by cognitive and emotional factors; attention, memory, and arousal all modulate our internal sense of time's passage (Droit-Volet and Gil, 2009). When we are engrossed in a task, our attentional resources are focused on the activity itself, not on monitoring time, leading to an underestimation of duration. Conversely, during periods of boredom or anxiety, heightened attention to time's passage can make it seem to slow down (Flaherty, 1991). The clock, however, continues its steady, indifferent march, creating the discrepancy we perceive. In the same spirit, subjective “speed” may partly reflect how readily experience is segmented into memorable events vs. how frequently one samples or consults an externalized temporal metric (Stojić and Nadasdy, 2024; Stojić et al., 2023).

The imposition of standardized time also has measurable psychological and physiological consequences. The constant awareness of a ticking clock can induce feelings of pressure, urgency, and stress, influencing decision-making and well-being (Hu et al., 2024; Qi et al., 2016; Sussman and Sekuler, 2022). More dramatically, societal conventions like Daylight Saving Time, which involve an abrupt, artificial shift of the clock, disrupt the alignment between our endogenous circadian rhythms and the external social clock. The imposition of standardized time and the biannual DST shift are linked to circadian misalignment and sleep loss, which are associated with mood disturbances and modest, short-term increases in acute myocardial infarction and ischemic stroke following the spring transition (Roenneberg et al., 2019; Rishi et al., 2020; Manfredini et al., 2019; Hurst et al., 2024; Sipilä et al., 2016). These effects show the deep-seated biological nature of our internal clock and its resistance to arbitrary, external manipulation. Standardized schedules also impact cognitive performance directly. Studies of students taking high-stakes exams show that performance declines steadily as the day progresses, a likely result of cognitive fatigue, and that even short breaks can significantly improve scores (Sievertsen et al., 2016).

The development of clocks, calendars, and now digital assistants can be understood as a form of cognitive offloading, the use of external tools to reduce internal mental load (Risko and Gilbert, 2016). By entrusting our schedules and reminders to an external device, we free up working memory and attentional resources for other tasks. This offloading can be seen as essential for navigating the temporal complexity of modern life. However, it may also come at a cost, potentially leading to a dependency on these external aids and a diminished capacity for internal time management when such tools are unavailable. At the same time, the turn toward externalized aids is not only a matter of load reduction. It may also reflect, and further reinforce, the uptake of an allocentric temporal framework in which activities are coordinated against shared, observer-independent reference systems (dates, timetables, and “objective” units) rather than solely against event-relative cues (Stojić and Nadasdy, 2024). On this view, later adolescence is a particularly important inflection point: as personal life becomes organized into an explicit narrative, individuals increasingly construct a mental timeline that supports reorganizing autobiographical memories along an abstract temporal axis, enabling more systematic alignment between “mind time” and “clock time” (Stojić and Nadasdy, 2024).

The role of both internal cognitive functions and external aids in maintaining temporal orientation is starkly illustrated in cases of neurological impairment. In some forms of dementia, individuals can experience dyschronometria, a breakdown in the ability to perceive the passage of time (Chen et al., 2019). They may confuse minutes with hours, days with seasons, or lose the ability to sequence events coherently. This condition, often associated with damage to the cerebellum and temporal lobes, shows our reliance on intact memory and attentional systems to construct a stable temporal world. The primary strategies for managing dyschronometria involve reinforcing external temporal scaffolds: maintaining strict daily routines, using large-print calendars, and employing specialized clocks that clearly state the day of the week and time of day (e.g., “Tuesday Morning”). This reliance on external aids in the face of internal deficits exemplifies the negotiation that characterizes the modern human temporal experience. Time perception in humans depends on a biological system rooted in events, emotions, and circadian rhythms. Superimposed upon this is a relatively recent cultural invention of abstract, standardized time, managed through an ever-growing array of technological tools. A complete model of human temporal cognition must account for our internal psychological processes and for how these processes interact with, and are often governed by, the external temporal frameworks that culture has constructed.

### 6.3 Establishing teMTT: transpersonal extended mental time travel

We introduce transpersonal extended mental time travel (teMTT) as a distinct, human-unique variant of mental time travel. teMTT arises when individual episodic simulation is systematically coupled with shared narratives and durable external records, producing temporal projection that is vicarious (reaching places and times no one present has experienced), collective (aligning many minds on common timelines and goals), and enduring (persisting across lifetimes). Storytelling is pivotal because language carries content beyond direct experience, from remote histories to counterfactual alternatives and fictional futures, allowing people who did not co-experience the events to coordinate on richly structured pasts and futures.

This builds directly on mind time as defined in the Introduction and on the developmental gains in Section 6.1 that make conventional time and external tools usable at scale. Mechanistically, teMTT builds on the same constructive–reconstructive operations that support individual MTT but adds dependable coupling between brains through shared representations. Listening to the same story can synchronize large-scale cortical dynamics across individuals, including default-mode regions implicated in episodic simulation; during narrative comprehension these patterns reorganize to track situation models over time, which provides a route for aligning internal event structure across listeners (Hasson et al., 2004; Simony et al., 2016). Communication can even transmit event representations from one brain to another: speakers' neural patterns during recollection are reinstated in listeners who later recall the events, indicating shared representational formats mediated by language and narrative structure (Zadbood et al., 2017).

These findings specify how individual simulations become aligned and shared, an essential step beyond solitary MTT for any temporally extended joint action. teMTT does not require words to operate. Wordless narratives in music, rhythm, gesture, image, and dance convey event structure, goals, and affect via temporal regularities and embodied cues. These media can synchronize attention and prediction across individuals and permit vicarious simulation even when propositional content is unknown, for example opera in an unfamiliar language, thereby coordinating mind time at group scale (Laland et al., 2016). In this sense, “story” denotes any structured sequence that supports situation-model construction and prospective inference, whether verbal or not, and whether performed live or preserved in artifacts.

External memory resources are the second pillar of teMTT because they extend remembering and imagining beyond biological limits. On the extended-mind view, reliable external resources such as speech, writing, images, maps, archives, clocks, books, and databases can function as integrated parts of cognitive processes when routinely used in the right way (Clark and Chalmers, 1998). Public artifacts provide lasting records that stabilize temporal information, enable correction and versioning, and permit recombination of episodes into new scenarios, supporting collective memory and cross-generational continuity (Roediger and Abel, 2015).

Social practices that standardize time, including calendars and clocks, allow large groups to synchronize plans over wide horizons and coordinate at scale (Zerubavel, 1982). Because information and plans persist outside individuals, ideas can be improved and recombined over long intervals, yielding cumulative cultural change and long-range coordination (Mesoudi et al., 2006; Caldwell and Millen, 2008; Dean et al., 2014; Muthukrishna and Henrich, 2016). Shared intentionality and coordinated narratives bind these resources to common purposes, aligning temporal reference frames, exchanging counterfactuals and conditional futures, and folding deep history into present decisions (Tomasello et al., 2005; Garcia-Pelegrin et al., 2021).

As such teMTT names the human-specific extension and sharing of mental time travel. Individual constructive–reconstructive processes are linked through language and other narrative media and stabilized by external records, which enables vicarious access to distant times, durable continuity across generations, and collective projection at societal scales. This account remains compatible with a comparative view in which other species exhibit elements of MTT, while reserving teMTT for the uniquely human coupling of simulation to public narrative and enduring records, with and without words. It also provides actionable design guidance for external time systems in §6: tools should represent event relations clearly, support correction and versioning, and remain compatible with clock time while respecting the event-relative structure people actually use in mind time.

## 7 Conclusion

This review advances five linked claims about temporal cognition. First, elements of temporal cognition can arise through convergent pressures across lineages, so similarities in performance should be expected where species face common ecological problems. Evidence from corvid caching and rodent temporal-order memory shows flexible recombination of bound content across delays and contexts rather than fixed routines (Clayton and Dickinson, 1998; Fortin et al., 2002; Babb and Crystal, 2005). This pattern aligns with comparative arguments for graded, task-dependent capacities rather than all-or-none taxonomic divisions (Suddendorf and Corballis, 2007; Allen and Fortin, 2013; Templer and Hampton, 2013).

Second, language, culture, and social organization set defaults for temporal orientation and interpretation in ways that are modest within individuals yet reproducible across tasks. Spatial–temporal metaphors, script direction, and gesture systems bias mental timelines and reference mapping (Boroditsky, 2001; Fuhrman and Boroditsky, 2010; Núñez and Sweetser, 2006). Grammatical aspect and “thinking-for-speaking” emphasize particular temporal diagnostics during encoding, which in turn nudges later recall and judgment (Slobin, 1996; Athanasopoulos and Bylund, 2013). These influences serve as priors that interact with goals and context rather than wholesale determinants of judgment.

Third, externalized time systems—clocks, calendars, schedules, and digital platforms—shape behavior by cooperating with or pulling against event-relative organization in mind. The historical shift from event-time to standardized clock-time created a durable coordination layer for large groups while introducing frictions where abstract time obscures dependencies among events (Mumford, 1934; Zerubavel, 1982; Levine, 1997). External tools function as cognitive offloading that reduces monitoring load and can improve prospective performance when cues are proximal to the action they target (Risko and Gilbert, 2016). Design matters: tools that surface event relations and decision points tend to support coordination better than tools that only expose uniform clock intervals.

Fourth, the adaptive value of memory lies in constructive use for prospection, and vulnerability to error follows from the same flexibility. The constructive episodic simulation account explains why the neural and psychological operations recruited for recollection also support simulation of novel futures by recombining stored elements (Schacter and Addis, 2007; Addis et al., 2009; Buckner and Carroll, 2007). Classic distortions such as suggestibility and source misattribution are predictable consequences of reconstruction and have patterned effects on temporal accuracy and ordering (Loftus and Palmer, 1974; Loftus, 2005; Johnson et al., 1993). Biological timing further modulates encoding, consolidation, and retrieval, producing lawful variation across circadian phase and sleep architecture (Gerstner and Yin, 2010; Rasch and Born, 2013).

Fifth, mind time names the internally constructed, event-relative structure that organizes recall and simulation. Event segmentation and situation-model dynamics supply the scaffolding for ordering, duration inference, and prediction during continuous experience (Zacks and Tversky, 2001; Baldassano et al., 2017). Timing behavior often reflects relative comparison rather than absolute metric readout, as shown by scalar variability and geometric-mean bisection across species and in humans (Gibbon, 1977; Gibbon et al., 1984; Wearden and Lejeune, 2008; Buhusi and Meck, 2005). These regularities clarify why modest changes to anchors, context, or goals alter perceived duration and order in systematic ways. It is for this reason that transpersonal extended mental time travel (teMTT) captures how communication and durable public records align many minds on shared pasts and futures across lifetimes. Narrative communication synchronizes situation-model dynamics across individuals, and speaker–listener coupling can transmit event representations that later guide recall (Hasson et al., 2004; Simony et al., 2016; Zadbood et al., 2017). External records and institutions stabilize temporal reference, support correction and versioning, and enable cumulative cultural change over horizons that exceed individual memory, which in turn expands the scale of coordinated planning (Clark and Chalmers, 1998; Roediger and Abel, 2015; Mesoudi et al., 2006; Muthukrishna and Henrich, 2016; Zerubavel, 1982).

The constituent literatures cohere under these claims. Hippocampal–cortical networks support recombination and monitoring for both recollection and simulation with content-sensitive specializations, consistent with default-mode and control contributions to construction rather than future-specific modules (Addis et al., 2007; Buckner et al., 2008). Linguistic and cultural practices shift priors for temporal reference and interact with reconstruction and biological timing to yield predictable biases in everyday decisions (Boroditsky, 2001; Droit-Volet and Gil, 2009). Comparative results are strongest when paradigms ensure bound delayed retrieval, sparse learning with generalization, flexible recombination under novel queries, and sensitivity to devaluation or transfer, which collectively argue for episodic-like construction over simple associative shortcuts (Clayton and Dickinson, 1998; Clayton et al., 2001a; de Kort et al., 2005).

Practical implications follow directly. Tools and institutions that present cues at the point of action, surface dependencies among events, and remain compatible with clock time while preserving event structure tend to improve prediction and coordination (Risko and Gilbert, 2016; Zerubavel, 1982). Educational settings can move learners from event-bound cues toward conventional units while keeping task structure visible, which supports the developmental shift in temporal control (Friedman, 2004; McCormack and Hoerl, 2017). Clinical and forensic practice can reduce distortion by limiting exposure to misleading post-event input and timing retrieval to biological rhythms that favor accuracy (Loftus, 2005; Rasch and Born, 2013). Organizations can improve handoffs by aligning cadence with natural event transitions and by maintaining current, shared representations that support versioning and correction (Roediger and Abel, 2015; Mesoudi et al., 2006).

A focused agenda can test and refine the five claims. Interfaces that foreground event relations can be compared with clock-first designs on plan quality, reconfiguration speed after perturbations, and resilience to misinformation. Developmental studies can track how the transition from routine-bound timing to conventional timekeeping changes the balance between flexibility and fidelity in recall and prospection (Friedman, 2004; McCormack and Hoerl, 2017). Comparative work can strengthen criteria that separate associative explanations from episodic-like construction through one-shot learning, transfer or devaluation, and novel query probes (Clayton and Dickinson, 1998; de Kort et al., 2005). Neurocognitive studies can relate replay, goal coding, and control to measurable gains in planning rather than only representational markers (Schacter and Addis, 2007; Buckner and Carroll, 2007). On this account, memory serves as a resource for building and negotiating futures at the individual level and, through teMTT, at the scale of communities and generations (Zadbood et al., 2017; Roediger and Abel, 2015), with historical practice as a clear case in which archives and narratives preserve past events so that they can inform future choices. In Carroll's terms, it is a “poor sort of memory that only works backwards” (Carroll, 2000, p. 174).
